# Co‐Production With Mental Health Family Carers: The Value, Outcomes and Benefits for Family Carers and Academic Researchers

**DOI:** 10.1111/hex.70657

**Published:** 2026-04-09

**Authors:** Caroline Walters, Melissa Petrakis, Sharon Lawn, Eileen McDonald, Carli Sheers, Hayley Solich, Jonathan Harms, Tony Stevenson, Melinda Goodyear, Marcelo Maghidman

**Affiliations:** ^1^ Department of Social Work Monash University Melbourne Victoria Australia; ^2^ Mental Health Service, St. Vincent's Hospital Melbourne Victoria Australia; ^3^ College of Medicine and Public Health Flinders University Adelaide South Australia Australia; ^4^ Lived Experience Adelaide South Australia Australia; ^5^ National Mental Health Consumer and Carer Forum Canberra Australian Capital Territory Australia; ^6^ Helping Minds Perth Western Australia Australia; ^7^ Mental Health Carers NSW Sydney New South Wales Australia; ^8^ Mental Illness Fellowship of Australia Brisbane Queensland Australia; ^9^ School of Rural Health Monash University Melbourne Victoria Australia; ^10^ Emerging Minds Hilton South Australia Australia

**Keywords:** collaborative research, community engagement, co‐production, family carer co‐researchers, mental health

## Abstract

**Introduction:**

Family engagement in mental health service reform, design, treatment, and research is highlighted as important within government and governance guidelines; however, inconsistencies of inclusion continue to be experienced and reported. Within mental health and community research there are calls for the adoption of participatory and co‐production methodologies.

**Objective:**

To add to the informed debate about patient and public involvement and engagement, this article explored the value, outcomes, and benefits of co‐production as a way of working with and elevating mental health family carers in research.

**Methods:**

Thirteen family carer co‐researchers participated in one of two focused‐conversations alongside two university academics, to reflect upon their experiences of working together across a 9‐month co‐produced research project. Co‐researchers were asked to consider experiences in relation to the meaning of participation, impacts of co‐production, and how this co‐produced research compared to involvement in other research. The co‐researchers were invited to participate in co‐analysing the focused‐conversations transcriptions, determining the findings, and authoring this paper.

**Results:**

Utilising an inductive approach to reflexive thematic analysis, insights into the values and benefits for family carers, academic researchers, research evidence, and outcomes were established. Themes developed about the values and benefits as well as the mechanisms of co‐production. Benefits were indicated for the researchers, participants, research process, evidence and outcomes. Mechanisms in relation to people's experiences of co‐production included: processes were different to other research experiences, practices were relational, and ethos was collegial and supportive. The value of co‐production was seen as privileging family carer voices, creating a sense of community and ‘sitting around the kitchen table’, leading to belonging and sharing within a space where people were safe, and participants felt validated.

**Conclusion:**

In recognising that co‐production is fundamentally different to traditional research, this paper revealed the value, outcomes, and benefits of processes, practices and ethos that engaged and elevated family carer leadership in research. Family carer co‐researchers experienced feelings of increased capability and identified that co‐production, is right if the end result resonates with the whole experience.

## Patient or Public Contribution

In recognition of the importance of family and unpaid carers' role within mental health care and support, this project was co‐produced with mental health family carers and service users from its inception. Their expertise in understanding the best ways to explore family carer experiences enabled nuanced findings to emerge. The two lead academic research partners have active experience of supporting people with mental health challenges. Through the phases of the original study, upon which the reflections occurred, lived‐experience expertise (family carers and service users) co‐designed and co‐facilitated the processes. This meant leading processes, such as in recruitment strategies, or acting as guides when considering areas of importance for research. Members of the original study steering group were invited and reflected upon their experiences within focused‐conversations. Co‐researchers read transcriptions of their focused‐conversations, identified key learnings, and were invited to co‐author this paper. The identified key learnings were developed and scrutinised from the perspective of the service users and carers in the project team. Co‐authoring meant identifying key areas for inclusion, editing the draft paper, choosing titles and suitable journals, and accepting the final version for publication.

## 1 Introduction

Internationally, the value and importance of engaging people at the centre of a research problem, concern or interest within research is recognised [1, 2, 3]. What is less known is the best methods for engagement; the who, when, how and how much. Traditional research and recruitment methods often fail to reach diverse and underserved populations [4]. Some challenges for community engagement include stigma, systemic barriers, lack of sensitivity and responsiveness, and reliance on academic and clinician‐led approaches [4]. The growth of patient and public involvement and engagement (PPIE) in research and service design is advancing awareness of the value and importance of people translating their experiences into better outcomes [5, 6]. However, challenges continue in raising the profile and epistemological impact of PPIE when strict hierarchies of what constitutes good scientific evidence exist [1], and there is ongoing evidenced scholarly resistance with studies discussing ‘research on’ people within a given cohort [7, 8, 9]. PPIE in mental health service treatment, design, and research is highlighted within government and governance guidelines, however, inconsistencies of inclusion continue to be experienced [10], alongside a paucity of guidance and reported studies [5].

Studies [11, 12, 13] recognise the value of mental health family carers (family members, friends, or supporters who provide unpaid care) in supporting the recovery journey and maintenance of wellbeing for people who experience psychological distress or utilise mental health services (described as service‐users within this paper). While it is known that care provision takes a “deep toll on the subjective wellbeing” of all carers ([14], p. 621), international studies noted higher family distress in families supporting people experiencing mental health challenges [15, 16, 17].

The Australian National Mental Health Commission, recognising the higher care load and potential increased distress, funded a study to understand the experiences of mental health family carers during the COVID‐19 pandemic. A research partnership was established between the National Mental Health Consumer and Carer Forum (NMHCCF), Monash University, and mental health community organisation representatives [18, 19]. The partnership, inclusive of two social worker academics (CW & MP), with their own family carer lived‐experience, co‐produced the research approach incorporating focus groups and a bespoke survey to collect data from mental health family carers in Australia. The national study found heightened levels of distress, increased caring responsibilities, and unmet needs [18, 19].

The ontological positioning of the Monash University Academics, and recognition of the expertise within the NMHCCF, led to a participatory, power sharing and transformative ethos adopted across all phases of the study. Given the partnership completed the study and submitted a government report within a 9‐month period, learnings from the co‐production process were seen as important.

### 1.1 Aim

To add to the informed debate about patient and public involvement and engagement, this article explored the value, outcomes, and benefits of co‐production as a way of working with and elevating mental health family carers in research. The paper answers the research question, what are the values, outcomes and benefits of co‐producing research with mental health family carers?

## 2 Materials and Methods

Studies to best identify and meet the needs of family carers should be determined by carers [20]. Often espoused as the ‘gold standard’ of evidence, randomised control trials may not be an appropriate methodology to seek to understand the complexity that carers and service users experience when interacting with mental health systems [21]. To promote power sharing within the research processes, a transformative ontology informed by feminist theory underpinned the study [22]. The exploratory qualitative study, occurred through two group focused‐conversations and utilised a narrative approach to understand people's experiences [23]. A full description of the materials and methods for the reflective process undertaken within this study by the research partnership, were reported in [24]. Adopting a group setting, facilitated members making sense of experiences together and built a collective view of the co‐production process, evidence and outcomes. The groups' narratives established findings around values, outcomes and benefits, alongside mechanisms of co‐production. The collegiality built across the research study was enhanced through the feminist methodology where the process of the research was viewed as important as the outcomes [22]. The two academic researchers, drew on their own family carer experiences to become part of the group, enabling collaboration and the establishment of practices that acknowledged the language and ways of working of the community to build sensitivity and empowerment.

### 2.1 Study Design

A purposive sample, being the members of the NMHCCF project steering group (PSG) and co‐facilitators of the original study focus groups [19], were invited by email to attend one of two online group focused‐conversations, inclusive of the academic researchers. Two experienced academics (MG & MM), independent of the original study, facilitated the conversations exploring project member experiences. Reflections regarding the national study's co‐production were considered from initiation to dissemination, and included the experiences of co‐authoring the report and peer‐reviewed publications. The questions for the facilitated conversations are available within Walters et al. [24] and covered concepts such as the overall experience of the project and co‐production, general and specific impacts, how could positive impacts be replicated, understandings of participation, and any further comments about experiences. The recorded discussions on the Zoom platform (https://www.zoom.com/) were transcribed using Otter (https://otter.ai/). The first author (CW), checked and deidentified the transcriptions, and returned the focussed‐conversation to the attendees for co‐analysis.

### 2.2 Participants

Six members of the PSG, and one non‐PSG focus group co‐facilitator attended the first group focused‐conversation, and six PSG members attended the second. The two academic researchers attended both. Co‐researchers lived in the states of New South Wales, Victoria, South Australia, Queensland, Western Australia and the Australian Capital Territory. There were diverse work and support roles represented, with both women (13) and men (2) participating in the conversations. A cross‐section of experiences and skills were self‐reported by the co‐researchers, including lived‐experience from both carer and service‐user perspectives, experiences of and supporting different mental health challenges and caring experiences, and ages. Co‐researchers included people who worked in grassroots organisations, had academic and research skills, led family carer or other mental health organisations, represented rural and regional geographic areas, and were from diverse cultural communities. As part of the focused‐conversation, the co‐researchers reflected on how the diversity informed the process and the project.

### Research Ethics

The study met the requirements for the National Statement on Ethical Conduct in Human Research and the Monash University Human Research Ethics Committee granted approval (Project ID:31516).

### 2.3 Data Analysis

Given the exploratory nature of the study, reflecting on the experiences of co‐researchers, an inductive approach to reflexive thematic analysis was undertaken [25]. Co‐researchers were provided a template to guide reflections and analysis of the transcription from their attended focused‐conversation. The template invited co‐researchers to identify quotes reflecting key messages conveyed within the transcripts, and reasons for their selection. The co‐researchers were also asked what messages were important for people/service providers/policy makers to learn from the co‐produced project methodology. Alongside the work of the co‐researchers, the corrected transcripts were uploaded by CW to Nvivo 14 (https://lumivero.com/product/nvivo/) for data management. An initial experiential thematic analysis was undertaken by CW, to identify semantic codes – grounding meaning within the expression of the participant's experiences [25]. The two experienced academics (MG & MM), who were not part of the original study and facilitated the focused‐conversations, were included in the data analysis, providing independent review.

The initial coding was discussed with MM and tentative themes grouped and formed. Early themes were explored, incorporating the submitted co‐researchers' reflections and written responses, and further developed through discussions with the other academic researchers (MP & MG). The review of the academic researchers' tentative themes with the co‐researchers' reflections to identify shared meanings and findings, provided further rigour and criticality leading to greater understanding of the themes and clarity in the wording that people felt were important.

[A note on identity, terms and language: whilst this paper refers to carers and service users, these are contested terms that both family members and people who experience challenges to their mental health may not relate to or use. In Australia ‘consumer’ is used in the place of ‘service user’. Consumer is used within identified quotes if this was the chosen term.]

## 3 Results

The analysis identified that the co‐researchers and academic researchers spoke of a number of beneficial impacts, as well as challenges, from co‐production in answering the research question, what are the values, outcomes and benefits of co‐producing research with mental health family carers? The mechanisms of co‐production within a national study were also spoken of strongly within the data. Subsequently, themes are presented in two groupings (Table 1); the first grouping of themes (five) in relation to the value and benefits of co‐production, then a second grouping of themes (three) addressing the mechanisms of co‐production. Given the exploratory and experiential nature of the study, as well as the importance of raising often marginalised voices, the findings are illustrated through the narratives of the researchers within the group focused‐conversations.

**Table 1:** Themes of the value and benefits of co‐production and mechanisms.

The value and benefits of co‐production	Mechanisms of co‐production
1. Partnership in Research Valued by Family Carer and Academic Researchers 2. A Sense of Safety and Welcome for Participants in COVID‐19 Study 3. Respectful, Accountable, and Open Research 4. Research Evidence was Nuanced, Enriched, and Accurate 5. Research Outcomes that Address Community Need	1. Processes (The Doing Of) were Different to Other Research Experiences 2. Practices (Ways of Working) were Relational 3. Ethos (Ways of Being Together) was Collegial and Supportive

### 3.1 Group 1 Themes ‐ the Value and Benefits of Co‐Production

Qualitative descriptions within the data highlighted the importance (value) and benefits of co‐production in relation to the experiences of researchers and participants, the processes, evidence and outcomes.

#### 3.1.1 Theme‐1: Partnership in Research Valued by Family Carer and Academic Researchers

Researchers spoke of co‐production fostering a partnership in research, which was differentiated from previous experiences of more extractive and transactional research. Partnering through building a sense of trust, was valued by the participants and researchers;

…building trust first, with folks who find it is often the experience …that they don't get heard. … within this particular project, we had one of the states where there was active awareness and therefore safe recruitment of people with an LGBTQI focus in what they wanted to share. There was an active space being made recruitment, engagement, and safe sharing with us in another state of people who are coming from an older age…

(Academic Researcher 2/[AR2])

Even co‐researchers who had been working in the field and were recognised leaders spoke of an increased sense of agency, empowerment, and support through the partnership (see Table 2). Being together and working collaboratively provided researchers with much‐needed respite from the isolation of being carers, and the holding of other people's distress (Increased during the COVID‐19 pandemic). They described a sense of trust in the process and in the relationships within the group, identifying the need for less emotional energy in being with people who understood your experiences, and not having to educate other members of the project team about shared experiences, nor to overcome power differentials or address hierarchical processes.

**Table 2:** Partnership in research valued by family carer and academic researchers.

The value and benefits of co‐production	Evidence (from focused‐conversation transcript)
Increased research team capacity and capability – through co‐learning from a diversity of expertise, knowledge, skills, and experiences	*The Project Steering Group, were not all carers, and they were not all lived‐experience. They were consumers and carers. There were people with research skills, there were people who lead various organisations around Australia. They understand the cohort. They represent rural remote, for example, people from other cultures. (FC2/CR4)*
Agency and ownership	*…as a group, [we] can take full ownership of the process. And therefore, we can carry that forward with full confidence that it's not something that's been done for us. It's something that we've been really embedded in and engaged in, … therefore, we own this project as a group. (FC1/CR2)*
A sense of belonging, strengthened relationships, and connection	*There's something very validating about being heard and being included and feeling like you belong. There [are] benefits in how we feel about and feel connected to the research and the data and what we can do with that, and how it enriches our understanding for our own practice, whatever we're doing, as lived‐experience workers, carers supporters out in the community, it's enhanced. (FC1/CR2)* *This group was really feeling of belonging. So, belonging, [in] the beginning, belonging, [in] the process and belonging [to] the outcome. (FC1/CR7)*
Less emotional energy required – not having to educate other project team members	*…we didn't have to… clear the clutter of not everyone being in the same space and some of the hierarchies and the power differentials… we all understood where we were at (FC1/CR4)*
Felt respected and active contributors	*…the pauses and the silences when people were able to reflect more deeply and think about what they wanted to say. And then they have the respect given to them to take that time and then to provide their response. (FC2/CR2)* *And there's been respect that even where there's been a difference of opinion, that we've been able to …with curiosity, investigate that difference, a sense of being respected in the process. (FC1/CR2)*
Support for leaders in the field	*The distress that the families, supporters and carers were having. Were, so extreme that it was having an impact on my own wellbeing, and trying to work with the service. And, with that balancing of wellbeing and risk, and all of these things and being so involved in that, … trying to get a feel for how pervasive this was across Victoria. And then it turned out that this wonderful project [was] going, so I was just able to kind of take a big sigh. (FC1/CR1)*
Validation of experience and capability	*For me, the benefit is the connection to the story and the evidence and what we can do with that, but also our own personal validating experience and growth in our understanding of how to do research and in a co‐design model. (FC1/CR2)*
Sense of accountability of academic researchers to co‐researchers	*I (CW) brought some technical skills, the ability to create a survey, to facilitate focus group, to do a literature review. But that also meant that it gave me a chance to do the work, then have check in points with the group. I saw it was my role to get things in order that could be presented to the group, to have their strong voices, and knowledge and expertise, look over what had been produced. (AR1)*
Safety and connection within new online approaches	*…it felt very safe for me, as a researcher and co‐facilitator to know that we had someone who knew most of the people who were going to be in the room. That there was going to be an element in there that it would be okay. (AR2)*

#### 3.1.2 Theme‐2: A Sense of Safety and Welcoming for Participants of COVID‐19 Study

The researchers spoke of benefits of co‐production processes on the experiences of the focus‐group and online survey participants, including increased safety and being welcomed. Through their observations of participants within the COVID‐19 study, co‐researchers felt the process was therapeutic, supportive, validating, and a space where people were safe and held.

I co‐facilitated a group, to just be in that group and to hear my story said by other people, or me sharing something of my story and other people nodding and knowing that they're having similar experiences, I think there was a very high therapeutic benefit through the process of gathering this information.

(FC1/CR2)

Further, in formulating the online survey research questions, based on a rapid scoping review undertaken by the academic researchers [26], the co‐production considered safety, including thoughtful use of language, such as wording for questions to minimise stigma, and the additions of options to account for the responsibilities of caring. Survey options included suggested times to take a break, reminders about the availability of support services, and the ability to leave and resume the survey at a later time.

### 3.2 Theme‐3: Respectful, Accountable and Open Research

The co‐researchers reported the experiences of co‐production processes were different to prior involvement in traditional research studies. Subsequently, a number of benefits to their experiences of the research process were identified through the adoption of co‐production (Table 3). The research was centred around family carers and noted as respecting the family carer co‐researchers' values, beliefs and ways of working. Ethical management of the process was a priority, with co‐researchers acknowledging their felt responsibility to ensure processes weren't harmful to the people they represented. Ethical practices were upheld through checking with the PSG and their guidance, especially in relation to language and non‐stigmatising practices. The diversity within the PSG enabled a wider engagement and inclusion of more diverse perspectives, alongside the capacity to draw upon different knowledges and skills of the PSG and academic researchers. The presence of known family carers co‐facilitating the focus groups, provided a sense of safety for the participants but also for the academic researchers in knowing that if the need arose there were supports available. All members of the research team brought an openness and flexibility. The academic researchers reported a sense of relational accountability to the PSG, which maintained motivation. An impact of importance to funding and authentic research completion was the efficiency and sustainability of the project. Through identifying common goals, shared meaning and understanding early, the project was able to progress more quickly.

**Table 3:** Respectful, accountable, and open research.

The value and benefits of co‐production	Evidence (from focused‐conversation transcript)
Ethical management of research –carried out in a safe respectful manner, with informed consent and genuine participation.	*I think, as an organisation, it was also important that we were giving access to the people that we serve, so it was important that we were able to also be there to ensure that the process was safe for them. And not that I had any concerns about that. But I think from the perspective of the organisation was also a good risk management strategy. (FC1/CR2)*
Flexibility and openness of research process	*You [PSG and academic researchers] weren't coming in with your preconceptions of what you wanted to know, you were much more open about what do we all want to know and how should we go about finding that out? (FC2/CR6)*
Family carer inclusion and engagement across all phases	*[making decisions] …even the tools, and in how we, …analysing the research findings and contributing to the final report, and even being able to co‐present at TheMHS, and it was amazing, …all part of every part of the research cycle. (FC1/CR6)*
Accountability through managing resources and openness to change	*We have finite resource of time, and of money. …sometimes that requires that one or two people might carry parts of the project. …if you are really working with true co‐design and co‐production, intention, everyone can be okay with that. It's when it's assumed to be okay for me to come and bring all of my idea and put it in front of you in that you're going to rubber stamp, that's not co‐production and co‐design. (FC1/CR2)*
Sustainability of group and research – group was sustained over an extended period, with ongoing relationships, shared roles including research dissemination and advocacy	*…the online environment gives you that capacity to reach and to have people from across Australia in the room at the same time …But it's also quite challenging to create a sense of community to keep people motivated or interested. So, they keep coming back for those, quite intensive slabs of meetings that you engaged in. …It's not just the outward facing, how you do your facilitation, but also how do you sustain people or a steering group, our group of people who are co‐designing the project processes together, how we get a sense of ourselves as a part of the community when we don't actually, have met them face to face? (FC1/CR5)*
Efficiency through common understandings and goals	*It [power differentials, hierarchies, different understandings] wasn't present in the process. So therefore, it wasn't taking up all the time, the precious time that was needed… We had already got past those things. (FC1/CR4)*

#### 3.2.1 Theme‐4: Research Evidence Was Nuanced, Enriched and Accurate

The researchers found co‐production influenced the research evidence to be more nuanced, meaningful, understandable and respectful in privileging the voices and perspectives of family carers within the research;

You actually are doing codesign, you're doing research in a different way that's giving you a different perspective. And that is the point in having lived‐experience led research, you are getting a different perspective, and privileging the kind of voice and perspective of people who don't always get heard, through research processes.

(FC2/CR6)

The PSG, holding a diversity of skills and experiences, were able to design the survey questions and focus group protocols to address more nuanced questions and experiences (Table 4). Identifying that their (PSG) experiences were found within the wider group of family carers, led to the co‐researchers evaluating the results were accurate and transparent. The co‐researchers noted that the voices of family carers across Australia were heard with ‘real understanding’, and that new knowledge was produced that may not have arisen from a standard existing survey. Researchers felt the diversity of family carer experiences gained through the greater reach of the national partnerships across 26 stakeholder organisations provided a breadth and depth of evidence which could be provided to these communities.

**Table 4:** Research evidence that is nuanced, enriched, and accurate.

The value and benefits of co‐production	Evidence (from focused‐conversation transcript)
Nuanced and enriched questions	*I think having the diversity [skills, roles, geographic and cultural diversity] on the Project Steering Group enriched the design for the questions that were used in the survey and in the focus groups. (FC2/CR4)*
New knowledge	*A benefit from genuine codesign is that you find out stuff, that you didn't know at the beginning, because you [have] actually undertaken genuine process of asking and finding out. (FC2/CR1)*
Diversity of evidence ‐ through different experiential, cultural and geographical lenses	*When we were talking about national lenses. It's not only federal and state, it is about mainstream and cultural diversity… we have some divisions when we are servicing the mental health of the mainstream, and with working with those from different cultural backgrounds. So, another plus of this research is having both perspectives, mainstream and cultural diversity. (FC1/CR7)* *The Project Steering Group, were not all carers, and they were not all lived‐experience. They were consumers and carers. There were people with research skills, there were people who lead various organisations around Australia. They understand the cohort. They represent rural remote, for example, people from other cultures. (FC2/CR4)*
Research results were reliable and validated by the co‐researchers reflecting their diverse experiences	*You've got the co‐design and co‐production, right if the end result resonates with the whole experience. It's not separate from it doesn't come out of left field; it actually is aligned. (FC1/CR1)*

#### 3.2.2 Theme‐5: Research Outcomes That Address Community Need

Co‐production led to a number of research outcomes (Table 5) beyond the requirements of the funding organisation, which was for a report with short‐term and long‐term recommendations to government. These further outcomes, were seen as important to raising the voice of family carers and increasing the exposure and ‘buy in’ into the report, alongside meeting and addressing community need and countering epistemic injustice.

**Table 5:** Research outcomes that address community need.

The value and benefits of co‐production	Evidence (from focused‐conversation transcript)
A meaningful record of family experiences through publications and the Government Report [27] available for advocacy purposes	*The gold that sits in the seam of this project, that Australian carers are on record, and have been heard by us. And we're going to amplify their voice as far as we can to drive change. (FC1/CR2)*
Understandable and respectful language – language utilised was common to families and respectful of participants (non‐stigmatising)	*…in reviewing the findings and talking that through and then [FC1/CR6] brilliant contribution of perspective to ensure that we were not further stigmatising consumers through the way we presented that information. (FC1/CR2)* *…when each of the areas of the survey was discussed, it really went into a lot of detail. A lot of discussion, line by line, question by question. Questioning the language, the tone, the assumptions, that were in the question. (FC2/CR2)*
Transparency and legitimacy of findings – findings are trusted by the community with honest reporting	*The work that's been produced throughout the process, has been brought with an openness for it to take a different direction if it needs to. I think that's been critical and was critical. In every stage and phase. … that's what co‐production, co‐design is, having that openness to the end result being a true reflection of the group process. (FC1/CR2)*
Authentic evidence for advocacy ‐ Story has weight – can see words in the final output that resonates with what was experienced	*It dawned on me how, if you participated in this, and you can see the words and thoughts and voices in the final result of what you heard as you were going through the process, or that you shared, then you know, you did something, right. (FC1/CR1)* *As [FC1/CR1] so beautifully put it, the final result has your footprint in there along with everyone else's footprint from the group (FC1/CR2)*
Capacity and skill bilding – growth in ability to do research	*For me personally, in any work that I would do in the future is how I learned from the process. (FC2/CR5)*
Address community need – countering epistemic injustice	*It's like emotional labour or something when you're part of a process or even read a paper, even ones that start to try and garner or capture experiential perspective, very often, the analysis is still from a provider perspective, or an overlay of previous assumptions. And that's incredibly painful. And so, it's epistemic injustice. So having not to deal with that was a huge benefit for this. (FC2/CR3)*

Findings from the national study were reported as having weight, being authentic evidence for advocacy. The final report [27], identified as a meaningful record of family carer experiences, was written and reviewed by all members of the PSG and submitted 31st August 2022. Alongside the academic researchers, PSG members presented the experiences of mental health family carers during the COVID‐19 pandemic at The Mental Health Services Conference (TheMHS) in October 2022, in Sydney, Australia. During this same period another member of the PSG (HS) presented findings at the Western Australia Carer's Week Conference. This was testament to the capacity and skill building, including growth in ability to do research through co‐production.

Ownership, through co‐production, added to the transparency, legitimacy and trust of findings by the community and government representatives. An infographic was created to provide quick access to the main findings for family carers (often identified as time poor), for information and advocacy purposes. The PSG and the academic partners worked together to organise the official launch of the report in August 2023. A 5‐min video was created by one of the PSG members (HS) with some of the family members who had been participants in the national study to illustrate key experiences within the report.

Members of the PSG were invited to co‐author papers, with two articles accepted for publication about family experiences during the COVID‐19 pandemic [18, 19]. Further papers were co‐authored with members of the PSG co‐reflecting on the process and establishing principles for co‐production with family carers [24].

### 3.3 Group 2 Themes – the Mechanisms of Co‐Production

Co‐analysing the transcripts of co‐researchers who held lived‐experience of the context and the process, developed three themes for the mechanisms of co‐production with mental health family carers; processes (the doing of) were different to other research experiences, practices (ways of working) were relational, and the ethos (ways of being together) was collegial and supportive.

#### 3.3.1 Theme‐1: Processes (The Doing Of) Were Different to Other Research Experiences

The co‐researchers reported the co‐produced research processes were family carer centred; with lived‐experience leadership and guidance from the beginning and across all phases of the project. Noted as different, was the responding to power from the beginning, research processes being adaptable to the needs of the family carer co‐researchers, embedding of lived experience, and approaches to focus group facilitation and preparation of the online survey (Table 6).

**Table 6:** Processes were different to other research experiences.

Processes were different	Evidence (from focused‐conversation transcript)
Acknowledging and responding to power from the beginning	*It's the structure of the groups, where the positional power sits, and it's paying attention to the early part of the process around co‐sensing, co‐creating, …making sure you have the right foundations to start the process and then constant attention to power. (FC2/CR3)*
Processes adapted to meet the needs of family carers	*I don't think there was any imposition of any agenda or set of assumptions. It was really, absolutely responsive to what was coming through the discussions. There were no limitations constraints, or we can't do it that way. (FC2/CR2)*
Embedding of lived experience	*…as a group, [we] can take full ownership of the process. And therefore, we can carry that forward with full confidence that it's not something that's been done for us. It's something that we've been really embedded in and engaged in, … therefore, we own this project as a group. (FC1/CR2)*
Presence of a family carer co‐facilitator identified as supportive	*The co‐facilitation model that we used, …was really key that family carers would be safe or more safe to disclose things that may be painful or surprise them later that they have ongoing repercussions of distress or concern (AR2)*

An example of a different process, were the decisions around co‐facilitating the focus groups;

I think it was helpful to the [focus] group, to see somebody that was familiar to them. Who was able to say, you know, this is the process we're about to engage in, and validating these people are going to treat you with respect and care for you through this process?

(FC1/CR2)

The co‐researchers reported how the openness and positioning of the academic researchers maintained the effectiveness (research addressed what was needed to represent the community), efficiency, and responsiveness of the research.

#### 3.3.2 Theme‐2: Practices (Ways of Working) Were Relational

The ways of working were reported as being relational compared to prior experiences of transactional research practices. Co‐researchers spoke of a sense of community amongst the project steering group members and working in partnership. All representatives of the stakeholder groups remained engaged across the 9‐month project. The early establishment of trust, through co‐sensing and co‐creating the purpose and aims of the project and collectively developing an understanding of the power relations. enabled shared roles to facilitate the progress, agency, and ownership (Table 7). Utilising people's strengths, led to relationships being multidirectional, and the relational ways of working continued through the PSG reaching out to their members and partners for recruitment and support within the research processes (focus groups and online survey). The relationship also motivated the academic researchers, who reported a sense of accountability to the PSG.

**Table 7:** Practices were relational.

Practices were relational	Evidence (from focused‐conversation transcript)
Sharing the load increased the sustainability of the project	*Having the core team to check everything with and we “co” did everything between us, whether we wrote an abstract… it wasn't a one person show, I think that contributed to the success… being able to stay on track, to being able to meet the key deliverables that we had, in terms of outputs for our funders… being able to share that load, and it not just all be on one person to have to think of everything, do everything and communicate and please everybody, to me that made it doable and sustainable (FC2/CR4)*.
Relationships were multidirectional	*…we have to have willingness, to collaborate and respect that sometimes some people may carry the intensive work, but they bring that intensive work with an openness for it could be completely changed …by the group. And that's the difference. (FC1/CR2)*
Relationships and connections assisted with greater reach for recruitment	*Through the partnership with 26 different stakeholders around Australia, the efforts that many of them such as the peaks did in the different jurisdictions, and others did to really pull in the people that they have relationship with, that they know about, to make it possible for them to have their needs for very diverse groups. (FC2/CR4)*
Motivation for the Academic Research Team	*Those checkpoints really helped. It helps to keep me [CW] motivated, to make sure that things were done on time. But I also became a learner through the process…being in rooms, and hearing things, that had been my experiences that was really valuable as well. (AR1)*

#### 3.3.3 Theme‐3: Ethos (Ways of Being Together) Was Collegial and Supportive

The underlying ethos of the research group was noted as respecting the values and beliefs of the family carer co‐researchers, which identified the ways of being together were collegial and supportive. Hierarchies and power differentials were absent, resulting in common understandings and goals. Co‐researchers expressed a sense of cultural humility and sensitive approaches to engagement, noting the use of pauses and silences to enable periods of reflection. Researchers acknowledged their positionality and respectful conversations led to supportive practices. Acceptance of diverse positions, ongoing connections across meetings, and opportunities to disseminate and present findings together, enhanced the sense of belonging. Mutuality ‐ a sense of being seen and heard through the sharing of common experiences ‐ was seen as an important part of the ethos of the project and healing for the participants within the focus groups.

I really enjoyed being part of the Project Steering Group, and it's really nice to be seen and heard, but from what people have been sharing, I just wanted to highlight the importance of mutuality. It's very validating when you hear someone share something that you can identify with or vice versa. I think it's an important part of the healing process.

(FC1/CR6)

### 3.4 Challenges of Participatory Research Methods Experienced by Mental Health Family Carers

The focused‐conversations revealed some challenges to co‐production, including the time for the COVID‐19 study to begin and be completed. Time delays arose through administrative sign‐off time for the grant and securing academic research partners who felt a ‘good fit’ for the research.

#### 3.4.1 In Waiting for Funding Administration Some of the Intensity of the Experiences Had Passed

In preparing for the national study and availability of funding, the more intense periods of restrictions for COVID‐19 had passed. The passing of time may have reduced the remembered intensity of the experiences for people. To reflect on the whole co‐production process, the focused conversations occurred after the launch of the report, potentially leading to lessened recollections of the process.

I congratulate everyone on that [comprehensive and careful approach]. It was pointed out to me how much time all this takes because, as I recall, as we went further and further on, we were getting further away from the peak times of being in lockdown, and the pandemic itself. So, you were constantly thinking now what was going on for me, or for us collectively, because we were moving further away from the experience of the pandemic and in Victoria, this unrelenting lockdown. So that would be my probably foremost comment that some of the height of that sensitivity and emotion, that you could bring to this was perhaps somewhat diluted.

(FC2/CR5)

The above concern suggests that funders should consider quickly identifying needs and supporting communities to act in emergent disaster situations.

#### 3.4.2 Co‐Produced Research May Take a Little Longer

With the choice of partners being held by the NMHCCF, this shortened the time required to build trust.

… that kind of is the point. If you're truly going to do the [co‐produced] research, you can act in a way that is really trying to shift the power balance. You can't do the research in the way that you always do the research. And you have to do it in a different way. That's what was done. And … that is going to take longer, and you can see you have to wait for everyone to talk and you have to draw out the perspectives.

(FC2/CR6)

The partnership continued evolving with guidance provided to the PSG during each phase, and ongoing encouragement and empowerment through the timely provision of information and resources by the academic partners for each individual to contribute and share their experiences.

…if someone is concerned about getting done quickly and close enough is good enough. And well, ‘you know, we've tended to do it that way before. So, let's just sort of move through it quickly’. That is the risk. That risk was avoided. And in fact, other processes were allowed to proceed, which did enable the momentum to pick up again, after the pauses and the silences when people were able to reflect more deeply and think about what they wanted to say.

(FC2/CR2)

As the researchers became more comfortable with each other, they were able to trust each other's roles in bringing information and work to the meetings.

## 4 Discussion

This paper explored how co‐production mechanisms, the processes, practices, and underlying ethos, added value and benefits for family carers, researchers, and research outcomes. Reflections within focused‐conversations generated learnings about the worth or importance (value) and benefits of co‐production with mental health family carers. Co‐production mechanisms included the processes (the doing of), practices (ways of working), and ethos (ways of being together). Co‐production principles focus on rebalancing power to partner with people at the heart of change or research [24].

### 4.1 The Value and Benefits of Partnership

This study highlighted the value of partnership including steps being considered and taken to authentically share power within co‐produced research, especially within powerful hierarchical systems such as health and academic institutions [28]. Empowerment has been noted through relationships [28, 29], and relational ways of working were shown to build community and maintain the sustainability of the group and research project.

Through the participatory principles described in Walters, et al. [24], including engagement from the beginning and the sharing of power across all phases, the research remained centred around family carers, illustrated lived‐experience leadership, and increased team capacity. Including family carers as co‐researchers and researching ‘with people’ and not ‘on them’, led to greater understanding of the problem and nuanced development of research questions to address the needs of the people at the centre of the research, cultural sensitivity and community safety.

However, partnerships and participatory research is not without its challenges, as it may require additional skills, funding and time, and considerations of balancing power and sharing governance to decrease the risk of tokensim [12]. The partnership developed across the 9‐months of the project and similar to other lived‐experience‐led projects [30], benefitted from shared values, goals, drive, and in‐depth understanding, which guided decisions on research questions, approaches, methods of engagement, safety, advocacy to government, and dissemination strategies. Mutual respect and recognising the strengths each person brought promoted open exchanges of knowledge and skills to expand the learnings. University partners provided research knowledge, skills, and work time to support the PSG to provide their expertise to discern and elevate the experiences of family carers and service users.

### 4.2 Respectful and Accountable Research Through Lived Experience Leadership and Guidance

Resourcing the project through a community organisation meant people at the centre of the research were supported to lead and participate over an extended period, and the choice of academic researchers aligned to community organisational values and ways of working. This reflects Scholz et al's [31]. discussion around the value and importance of patient and public participation moving to lived‐experience leadership. Leading from family carer and consumer lenses, co‐researchers combined and further developed expertise gained across personal, informal, professional, advocacy and organisational experiences. Subsequently, research processes became more responsive to the rapid changes for engagement occurring across the pandemic period.

Similar to Gillard et al.'s [1] study, who worked with people utilising their expertise by experience within research, the research process changed, becoming non‐hierarchical, collaborative, transdisciplinary, producing knowledge that was more socially accountable and representative of the community. A benefit for this project was the PSG were experienced and paid members of family carer and consumer organisations, with well‐established networks and active memberships from eight states and territories in Australia. Members had been working together on a number of issues to represent members over months, and for some, years, which also meant they may not experience some of the early challenges of co‐production. Challenges to co‐production identified by Gee, et al. [32] included individual level barriers being related to fatigue, stress, the high level of commitment and skills required for engagement, changes in life circumstances, not being provided with information in advance of attendance at meetings, and uncertainty about times, purpose and directions of meetings. However, the lived‐experience expertise, depth and breadth of connections across the PSG, enabled drawing upon the co‐researchers' experiences and enhanced the recruitment processes. Given the move to online methods for engagement, during the pandemic, working with co‐researchers known to the community of the participants increased safety and connection. These factors led to greater efficiencies, accountability, and rigour.

In dismantling some of the academic norms associated with research [33], the co‐facilitation of focus groups was again seen as bringing support and safety for family carer participants. Coming together as a group during the period of COVID‐19 ‘lock downs’ resulted in participants experiencing a sense of connection and support whilst sharing their experiences, which was described as therapeutic. In recognising that “we should not shy away from distress and sadness in research” ([33], p. 155), it is important to hold and support participants through their experiences of vulnerability – the co‐facilitation process was able to do this. The complexity and richness of experiences were able to be sensitively documented within the government report and academic papers.

### 4.3 Sensitivity to Communities and Culture

A further strength of co‐production was additional cultural sensitivity and community safety through inclusion of people from diverse groups at the centre of the research interest, which is often a challenge in health research, especially noted with the intersectional relationships between social determinants of health and mental health [5, 30, 34]. The addition of diverse voices and perspectives helped frame the final report. Family carer experiences were acknowledged without stigmatising service users by emphasising the strength of carer experiences resulted from poor systemic responses to the pandemic and not increased demands from the people they supported or were in a care relationship with. The PSG spoke of a sense of ownership for the research and the outputs and outcomes. The recommendations and evidence of the experiences of family carers, was validating, but more importantly gave PSG members confidence in being able to perform their leadership and advocacy roles.

### 4.4 Epistemic Justice Through Research Evidence

The resulting benefits in the sharing of power and knowledge within co‐production, increased attunement and appropriateness of the research findings to cultural contexts, empowered communities, and facilitated knowledge translation into recommendations for policy changes, reflecting Jagosh et al. [35] findings. The co‐produced national study successfully generated outcomes with key and authentic insights and recommendations for positive change that resonated with family carers [18, 19].

In partnering with researchers with lived‐experience, academic researchers should have a core aim for social change [36]. Compatibly, social justice is a core value of the lived‐experience movement and peer workforce [37]. Okoroji et al. [36] go further to say that if the aim of the research process is not for social change, then “the research process can be oppressive in its own right” (p. 4).

Through co‐producing the research topic and question, working together in refining methods of data collection, considering together how analysis occurred and learnings presented and shared, a number of key benefits arose in the quality of the evidence produced. The elevated inclusion of lived expertise, meant establishing the group and research questions were found to be efficient based on common goals, enabled recruitment through reach of the stakeholders, and the findings were relevant to the end users. The findings were meaningful to family carers, representing and privileging intersectional perspectives, and situated within the reality that they experienced. Intersectional perspectives are seen as one of the strengths of co‐production and working with people in research and knowledge generation [33]. The research evidence, in the original study, gained through co‐production led to the raising of family expertise in advocacy and subsequently 10 recommendations to governments within Australia to enact system change. The power achieved through connections and relationality were elevated within the co‐production process; different communities, skills, backgrounds, and roles were able to work together in shared endeavour and commitment, and achieved a more authentic and meaningful outcome.

### 4.5 Limitations

Limitations arose within the context of the COVID‐19 pandemic, and the delay for this reflective study. Prior to the national study, some members of the PSG had worked together and therefore were known to each other, which may be seen as a strength and a limitation. The drivers of the project for the NMHCCF invited participants into the PSG and therefore may have limited the diversity of representatives. While steps were taken to include members from multicultural and First Nations communities, numbers were lower than hoped for.

An honorarium was offered for members of the PSG to participate in the focused‐conversations. However, funding was not available to pay people to co‐author papers reflecting on the process. Only 13 of the 23‐member PSG attended the focused‐conversations, which represented most states, and family carer and service user perspectives, but may not represent all members' views or perspectives. Due to the busyness of reform and policy changes, the launch of the report was in August 2022, a year after the final report from the study was submitted in August 2021. Consequently, reflections occurred later after the completion of the research process than desired, but were designed to incorporate all aspects including dissemination. The delayed reflections may mean that all the essential components of the process were not captured. The academics who facilitated the focused‐conversations did not openly identify as having family carer experiences, which may have influenced the environment and analysis.

## 5 Conclusion

In drawing on critical, feminist, and empowerment theories, this paper addresses the influence of privilege, society, and politics in shaping the nature of reality. The value and benefits of academic researchers working alongside mental health family carers was demonstrated. Reflections on co‐production in research identified it was collaborative, acknowledged power and social justice, and supported community advocacy. With the axiological foundation being relational, and in working together to generate knowledge, a number of benefits to the research were achieved. Benefits included knowledge that was more socially accountable and representative of the community; increased safety and connection across the project, including when responding to the rapid changes for engagement during the pandemic period; identified efficiencies, authentic research that reflected the community interests; and documenting the complexity and richness of experiences.

Many family carers have experienced research ‘on’ families. The researchers endeavoured to change this and find an approach to working ‘with’ families. It is hoped this approach will influence future research and advocacy practices.

### 5.1 Relevance for Practice

The academic researchers came without preconceptions; they came with a sense of curiosity, adopting a responsive and flexible way of researching. Whilst not a necessity, academic researchers being members of the community enabled a faster and deeper understanding of experiences. Working with lived‐experience researchers promoted recruitment and support of participants. Improved research design and questions led to authentic and impactful findings for communities and report submissions for system change.

The challenges of becoming the carer or supporter of a person with psychological distress or a mental illness cannot be understated. Employed, job secure researchers must pave the way for engagement and participation for busy and at times exhausted family carers to partner in research. Academic researchers must consider barriers and facilitators for participation, and be advocates to support authentic co‐production.

### 5.2 Future Work

Action on the findings, recommendations and next steps from this research and the underlying national study depend on grant and funding practices recognising the value of mental health family carers in research. Supporting community and lived‐experience people accessing research opportunities, requires flexibility in tenders and grants for sufficient detail of processes, which may include considerations for research proposals with an overarching structure with limited detail to allow co‐production of the entire process. Similarly, ethics structures lack acknowledged processes for co‐researchers who may be participants. People are defined within boxes. Forms insist co‐researchers be ‘othered’ as ‘participants' who need to give consent. To establish research processes that evidence lived‐expertise leadership – research and publishing practices should support and be transparent in identifying people with lived‐experience as authors.

Policies and research focus should move from assisting family carers within care and support roles, to upholding the human rights of family carers [38]. Co‐production can uphold family carer human rights, through research and policies that support family carers returning to participating in their life prior to the care role.

## Author Contributions

**Caroline Walters:** conceptualisation, investigation, funding acquisition, writing – original draft, methodology, validation, writing – review and editing, software, formal analysis, project administration, data curation. **Melissa Petrakis:** conceptualisation, investigation, funding acquisition, writing – original draft, methodology, validation, writing – review and editing, formal analysis, supervision. **Sharon Lawn:** conceptualisation, investigation, funding acquisition, methodology, writing – review and editing, formal analysis. **Eileen McDonald:** conceptualisation, investigation, funding acquisition, methodology, writing – review and editing, formal analysis. **Carli Sheers:** conceptualisation, investigation, funding acquisition, methodology, writing – review and editing, formal analysis. **Hayley Solich:** conceptualisation, investigation, funding acquisition, writing – review and editing, methodology, formal analysis. **Jonathan Harms:** methodology, writing – review and editing, formal analysis, investigation. **Tony Stevenson:** methodology, writing – review and editing, formal analysis, investigation. **Melinda Goodyear:** conceptualisation, investigation, methodology, writing – review and editing, formal analysis, supervision. **Marcelo Maghidman:** conceptualisation, investigation, methodology, writing – review and editing, formal analysis, supervision.

## Ethics Statement

The Monash University Human Research Ethics Committee was satisfied the study met the requirements for the National Statement on Ethical Conduct in Human Research and granted approval (Project Id 31516). Participants received an explanatory statement attached to the invitation for participation. At the commencement of the focused‐conversation, participants were asked for their verbal consent to participate and have the meeting recorded.

## Conflicts of Interest

The authors declared no potential conflicts of interest with respect to the research, authorship, and/or publication of this article.

## Data Availability

The data that support the findings of this study are available on request from the corresponding author. The data are not publicly available due to privacy or ethical restrictions.
